# The Chemistry of Polydopamine Film Formation: The Amine-Quinone Interplay

**DOI:** 10.3390/biomimetics3030026

**Published:** 2018-09-13

**Authors:** Maria Laura Alfieri, Lucia Panzella, Stefano Luigi Oscurato, Marcella Salvatore, Roberto Avolio, Maria Emanuela Errico, Pasqualino Maddalena, Alessandra Napolitano, Marco d’Ischia

**Affiliations:** 1 Department of Chemical Sciences, University of Naples Federico II, Via Cinthia 4, I-80126 Naples, Italy; marialaura.alfieri@unina.it (M.L.A.); panzella@unina.it (L.P.); alesnapo@unina.it (A.N.); 2 Department of Physics “Ettore Pancini”, University of Naples Federico II, Via Cinthia 4, I-80126 Naples, Italy; oscurato@fisica.unina.it (S.L.O.); marcella.salvatore@fisica.unina.it (M.S.); pasmad@fisica.unina.it (P.M.); 3 Institute for Polymers, Composites and Biomaterials, National Council of Research of Italy (IPCB-CNR), via Campi Flegrei 34, I-80078 Pozzuoli, Italy; roberto.avolio@ipcb.cnr.it (R.A.); mariaemanuela.errico@ipcb.cnr.it (M.E.E.)

**Keywords:** polydopamine, polymerization, film, quinone, periodate, amines

## Abstract

Despite extensive investigations over the past decade, the chemical basis of the extraordinary underwater adhesion properties of polydopamine (PDA) has remained not entirely understood. The bulk of evidence points to PDA wet adhesion as a complex process based on film deposition, and growth in which primary amine groups, besides catechol moieties, play a central role. However, the detailed interplay of chemical interactions underlying the dynamics of film formation has not yet been elucidated. Herein, we report the results of a series of experiments showing that coating formation from dopamine at pH 9.0 in carbonate buffer: (a) Requires high dopamine concentrations (>1 mM); (b) is due to species produced in the early stages of dopamine autoxidation; (c) is accelerated by equimolar amounts of periodate causing fast conversion to the *o*-quinone; and (d) is enhanced by the addition of hexamethylenediamine (HMDA) and other long chain aliphatic amines even at low dopamine concentrations (<1 mM). It is proposed that concentration-dependent PDA film formation reflects the competition between intermolecular amine-quinone condensation processes, leading to adhesive cross-linked oligomer structures, and the intramolecular cyclization route forming little adhesive 5,6-dihydroxyindole (DHI) units. Film growth would then be sustained by dopamine and other soluble species that can be adsorbed on the surface.

## 1. Introduction

Despite extensive investigations over the past decade [1], the structural and chemical basis of the exceptional underwater adhesion properties of polydopamine (PDA) has remained in part uncharted. A number of structural studies, starting from 2012, have concurred to suggest that PDA is made up of a complex ensemble of structural units, including uncyclized amine-containing moieties and cyclized indole-type units, linked together via both covalent bonds, and non-covalent interactions, such as hydrogen bonding, cross-links, stacking, catechol/semiquinone/quinone and π-cation interactions [2,3]. Thus far, the strong attraction between the poly(catecholamine) layers has been attributed to surface salt displacement by the primary amine and π-cation interactions between the protonated amine groups and aromatic rings, whereas the chelating properties of the catechol functionalities have been implicated in the adhesion properties toward specific substrates [4,5]. The central role of amine groups in the adhesion and cohesion processes underlying PDA film deposition and growth is supported by various lines of evidence. Previously, we reported that replacing the amine group of dopamine with a hydroxyl group, as in hydroxytyrosol, results in the formation of polymeric materials completely devoid of adhesion and film forming properties [6]. In a subsequent study it was shown by means of nanomechanical measurements using a surface forces apparatus that the adhesive strength between poly(catecholamine) layers is 30-times higher than that of poly(catechol) coatings [4]. In a related study it was demonstrated that addition to the PDA reaction mixture of a long chain diamine, hexamethylenediamine (HMDA), results in a film rich in amine groups with high cross-linking and low tendency to swell [7]. Most interestingly, other studies demonstrated that HMDA can induce film deposition and coating from a variety of catechol-type substrates that otherwise do not form films. Substrate-independent film deposition was thus reported from gallic acid [8], caffeic acid via benzacridinone scaffolds [9], and catechol [10]. 

Another relevant issue is whether other structural units and functional groups in addition to amines, especially cyclized 5,6-dihydroxyindole (DHI) units, play a role in PDA film deposition and coating. This issue was addressed in a recent study [11] showing that pure DHI, under the same conditions used for PDA deposition, does not lead to adhesive films by dip-coating at pH 9.0 despite massive precipitation of eumelanin-type polymer. In addition, in the same study it was shown that PDA films do not present main peaks in common with DHI-melanin on matrix-assisted laser desorption/ionization–mass spectrometry (MALDI–MS) analysis, suggesting the lack of pure DHI-based structures. 

Although there is to date enough evidence indicating that primary amine groups, besides catechol moieties, play a vital role in PDA wet adhesion, the detailed interplay of chemical interactions underlying the dynamics of film formation has remained little understood. Important insights into the mechanisms underlying PDA film deposition can be obtained by studies of concentration dependence. Early studies on this aspect [12,13] showed that dopamine concentration markedly affects film thickness and the rate of film deposition. Increasing dopamine concentration increases the maximal thickness, which in turn is correlated with greater root-mean-square (RMS) roughness. During PDA deposition nanometer-scale structures are rapidly produced on the surface and deposition rate decays exponentially due probably to the evolution of PDA aggregates. Increasing the oxygen concentration of the solution leads to a significant decrease in roughness as compared to that observed with ambient oxygenation [14]. 

Notably, no coating formation was reported at low dopamine concentrations (<0.1 g/L) or when the substrate was immersed after the process of PDA formation was completed (e.g., after 24 h autoxidation). It was concluded that PDA film formation follows two different mechanisms with different regimes, namely film deposition and film growth [12]. These results raised a number of important mechanistic issues concerning: (1) Which of the mechanisms underpinning film deposition are failing at low dopamine concentrations? (2) What are the species that are responsible for film deposition? (3) Is the film growth process based mainly on cohesive interactions, distinguishable and different from the film deposition process, relying on adhesive interactions? 

The aim of the present study is to address in a systematic manner these central issues by means of a chemical approach based on: (a) A re-examination of the dependence of film formation on dopamine concentration; (b) an analysis of the evolution of the film-forming properties of PDA with the course of dopamine oxidation; (c) the effect of the fast generation of dopamine quinone on the two phases of film deposition and growth; and (d) the effect of added amines on film deposition. Based on the preliminary results of these experiments, an improved chemical model for PDA film formation is proposed, in which the two processes relating to film deposition and growth are interpreted in terms of concentration-dependent intermolecular amine-quinone chemistry and of subsequent monomer adsorption on the deposited layers, respectively.

## 2. Materials and Methods

### 2.1. Materials

All reagents were purchased from commercial sources (Sigma-Aldrich, Milan, Italy; and Carlo Erba, Milan, Italy) and used without further purification. Quartz substrates were cleaned by soaking in piranha solution (96% H_2_SO_4_/30% H_2_O_2_ 5:1 *v*/*v*) overnight, rinsed with distilled water and dried under vacuum.

### 2.2. Synthesis of PDA and General Procedure for Substrate Coating

Polydopamine was prepared as previously reported by autoxidation of dopamine hydrochloride (100 mg, 0.5 mmol) in 0.05 M carbonate buffer (pH 9.0), or 0.05 M phosphate buffer (pH 9.0), or 0.05 M Tris buffer (pH 8.5) (final concentration 1 mM or 10 mM), under vigorous strirring. 

Quartz substrates were dipped in the autoxidation mixtures after complete dissolution of the catechol. After 24 h, substrates were rinsed with distilled water, sonicated in methanol/water solution 1:1 *v*/*v*, and air-dried. The coated substrates thus obtained were analyzed by ultraviolet–visible (UV–vis) spectrophotometry on a Jasco V-730 instrument (Jasco, Lecco, Italy). When required, after 24 h the reaction mixture was acidified to pH 2 with 4 M HCl, centrifuged at 5488× *g* at 4 °C (Thermo Fisher Scientific, Rodano, Italy), and the precipitate washed three times with water and lyophilized to collect the dark pigment (45% *w*/*w* yield).

In separate experiments, to investigate the evolution of film forming during PDA synthesis from 10 mM dopamine in carbonate buffer (pH 9.0), quartz substrates were dipped in the solution for the appropriate amount of time according to the experiment (between 1 and 24 h), and then rinsed and analyzed as described above. 

### 2.3. Synthesis of PDA-Amines Films

To a 1 mM solution of the appropriate amine (HMDA, ethylenediamine, 1,10-diaminodecane, 1,12-diaminododecane, 1,4-diaminobutane, butylamine or dodecylamine) in 0.05 M carbonate buffer (pH 9.0), dopamine hydrochloride was added under vigorous stirring in a 1:1 molar ratio. Quartz substrates were dipped into the reaction mixture and left under stirring for 24 h, then rinsed with distilled water, sonicated, dried and analyzed as above.

### 2.4. Synthesis of PDA-Periodate Films

Dopamine hydrochloride (100 mg, 0.5 mmol) was dissolved in 0.05 M carbonate buffer (pH 9.0) (final concentration 10 mM) followed by sodium periodate in a 1:1 molar ratio. The reaction mixture was left under vigorous stirring for 24 h. The quartz substrates were dipped in the solution for the appropriate amount of time according to the experiment (between 5 min and 24 h). They were then rinsed with distilled water, sonicated, dried and analyzed as above. 

After 24 h the reaction mixture was acidified to pH 2 with 4 M HCl and the dark pigment was collected by centrifugation at 5488× *g* at 4 °C, washed three times with water, and lyophilized (90% *w*/*w* yield).

### 2.5. Film Growth

Polydopamine films obtained as described above by periodate oxidation of 10 mM dopamine for 1 h were immersed into solutions of 0.1 or 1 mM dopamine in 0.05 M carbonate buffer at pH 9.0 and left under stirring for 3–6 h. The substrates were rinsed and analyzed as usual.

### 2.6. Atomic Force Microscopy and Micro-Raman Analysis

The combined atomic force microscopy (AFM) and micro-Raman analysis were conducted with the integrated apparatus Alpha300 RS (WITec, Ulm, Germany). The system can be switched at will between AFM and confocal micro-Raman configurations, allowing a combined topographical and spectral characterization of a specified microregion of the sample. The samples topographies were studied by AFM in intermittent contact (AC) mode using a cantilever with 75 kHz resonant frequency. For the micro-Raman analysis, a laser beam at λ = 488 nm was used as excitation light source. The beam was focused onto the sample surface by means of a 50× microscope objective (numerical aperture (NA) of 0.75) working in epi-illumination mode. The diffraction-limited focused spot in the objective focal plane had a full width at half-maximum (FWHM) of approximately 320 nm. The light backscattered from the sample was collected by the same objective, and sent to the spectrograph through a confocal optical collection path. The samples analysis was conducted in the microregions marked by the colored squares in the optical images of the films. The AFM images correspond to an area of 25 × 25 μm^2^ (yellow square). For the micro-Raman imaging, the samples were scanned over the area of 18 × 18 μm^2^ indicated by the red squares. The scanning step was set at 320 nm in order to match the FWHM of the diffraction-limited focused laser spot. The Raman spectra results from 500 ms acquisition time, while the Raman images of the analyzed regions were reconstructed integrating for each scanned position the Raman signal in a spectral window of 140 cm^−1^ in width, centered at the 1584 cm^−1^ peak.

### 2.7. Solid State Nuclear Magnetic Resonance 

Solid state nuclear magnetic resonance (NMR) spectra were recorded on a Bruker Avance II 400 spectrometer (Bruker Corporation, Billerica, MA, USA) operating at a static field of 9.4 T, equipped with a Bruker 4 mm magic angle spinning (MAS) probe. Samples were packed into Bruker 4 mm zirconia rotors sealed with Bruker Kel-F caps. The spinning speed was set at 10 and 6 kHz for ^13^C and ^15^N NMR experiments, respectively. Cross polarization (CP) spectra were recorded with a variable spin-lock sequence (ramp CP-MAS), and a relaxation delay of 4 s; a ^1^H π/2 pulse width of 3.0 μs was employed and high-power proton decoupling was applied during acquisition. For ^13^C spectra, the contact time was set to 2 ms and 20,000 scans were recorded per each sample. Spectra were referenced to external adamantane (CH_2_ signal 38.48 ppm downfield of tetramethylsilane (TMS), set at 0 ppm). For ^15^N spectra, the contact time was set to 1.5 ms and 80,000 scans were recorded. Spectra were referenced to external glycine (amine signal 32.6 ppm downfield of ammonia, set at 0 ppm). 

## 3. Results

### 3.1. Concentration Dependence

**Figure 1:**
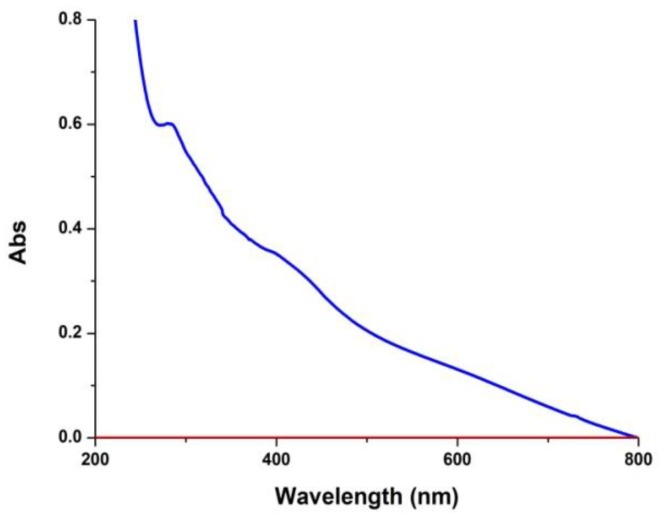
Ultraviolet–visible (UV–vis) absorption spectra of quartz substrates subjected to dip-coating with dopamine 1 mM (red trace) and 10 mM (blue trace).

In a first series of experiments the dependence of PDA film deposition on the initial dopamine concentration was re-examined using carbonate buffer at pH 9.0. For the purposes of these experiments, the final absorbance on quartz was taken as a rough index of the degree of coating deposition. The results showed that at 1 mM concentration (or lower) dopamine autoxidation in carbonate buffer does not result in ultraviolet (UV)-detectable coatings (Figure 1). 

**Figure 2:**
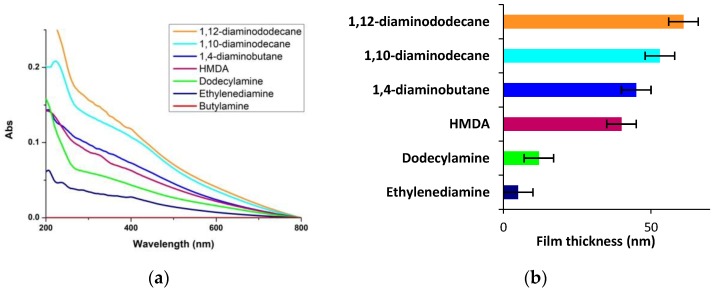
Ultraviolet–visible (UV–vis) and film thickness analysis. (**a**) UV–vis spectra of quartzes dipped into 1 mM solution of dopamine in 0.05 M carbonate buffer (pH 9.0) in the presence of equimolar amounts of various amines over 24 h. (**b**) Average film thickness as determined by atomic force microscopy (AFM). Data are shown as mean ± standard deviation (SD) of three independent experiments.

Changing the buffer from carbonate to Tris or phosphate did not lead to detectable film deposition from 1 mM dopamine. Likewise, addition of 1 mM sodium periodate, to induce the fast formation of the *o*-quinone, was not effective in inducing UV–vis-detectable film deposition with 1 mM dopamine. Notably, however, when HMDA or other amines were added to 1 mM dopamine at pH 9.0, variable levels of coating formation were observed (Figure 2). 

Data in Figure 2 show that a long aliphatic chain and two amine groups are important structural determinants for film deposition, and the longer the chain, the higher the effect. Consistent with this conclusion a short chain monoamine (butylamine) proved not effective at inducing film deposition, whereas the highest extent of coating was induced by 1,12-diaminododecane, based on UV–vis absorption and AFM measurements (see Supplementary Figure S1). 

**Figure 3:**
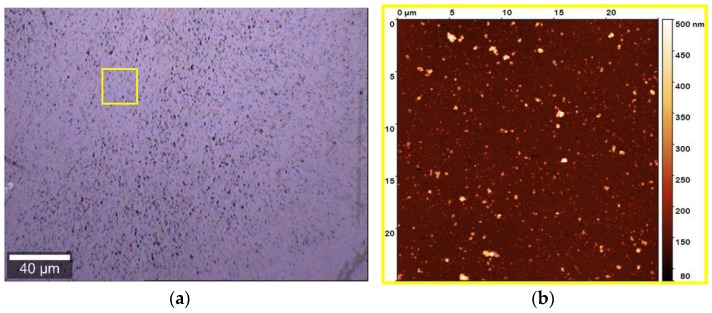
Atomic force microscopy (AFM) analysis of the polydopamine (PDA) film obtained in the presence of hexamethylenediamine (HMDA). (**a**) Bright-field image of the investigated sample region collected by 20× microscope objective. (**b**) AFM image of the area indicated by the yellow square in the optical image. Film thickness: 40 ± 15 nm.

Morphology and thickness of PDA films obtained in the presence of HMDA at 24 h oxidation time, was investigated by means of AFM. The mean estimated thickness was 40 nm. As visible from the optical and AFM images (Figure 3a,b), a uniform film adhesion and good grain distribution and dispersion was achieved. 

### 3.2. Temporal Evolution of Film Forming Properties and the Role of Dopamine Quinone

**Figure 4:**
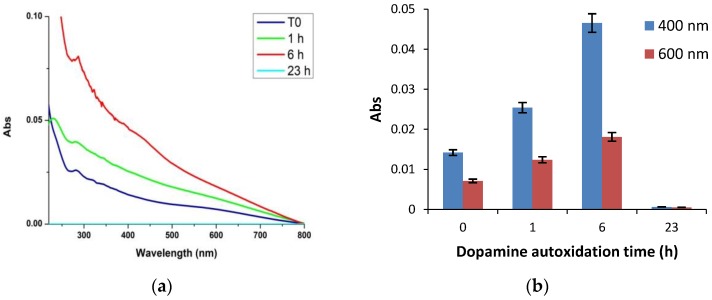
Kinetics of polydopamine (PDA) film formation. (**a**) Evolution of the ultraviolet–visible (UV–vis) spectra of PDA film formation with quartz substrates dipped into the mixture for 1 h at different reaction times. (**b**) Absorbance of the films at two selected wavelengths. Data are shown as mean ± standard deviation (SD) of three independent experiments.

In further experiments the evolution of the film-forming properties of PDA were investigated during the polymer synthesis from 10 mM dopamine. To this aim quartz substrates were dipped for 1 h in the dopamine solution undergoing autoxidation in carbonate buffer at pH 9.0 at different times after oxidation had started. The results showed that formation of the coating species is highest in the early hours and becomes negligible after 23 h, indicating that film formation properties are specifically associated with early intermediates in dopamine autoxidation and are virtually quenched at the end of the process when extensive aggregate precipitation occurs (Figure 4).

**Figure 5:**
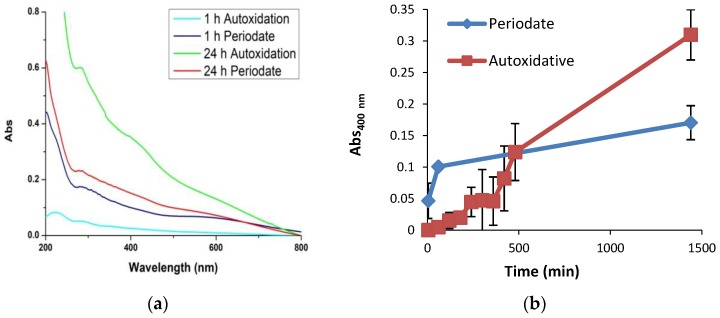
Kinetics of polydopamine (PDA) film formation: autoxidation vs. periodate. (**a**) Evolution of the ultraviolet–visible (UV–vis) spectra of PDA film formed by dopamine autoxidation or periodate-induced oxidation. (**b**) Time course of 400 nm absorption development for PDA film formed by dopamine autoxidation (red line) vs. periodate induced oxidation (blue line). Data are shown as mean ± standard deviation (SD) of three independent experiments.

To assess the role of dopamine quinone in film formation, in subsequent experiments film deposition from 10 mM dopamine at pH 9.0 was investigated using 10 mM periodate to induce fast quinone formation. The results reported in Figure 5 showed that PDA film deposition proceeds at much faster rate in the presence of periodate compared to autoxidation. Notably, however, the final absorbance of the film at 24 h proved to be considerably less intense than that of the autoxidation process. It is concluded that fast quinone formation accelerates the initial coating deposition, but prevents the films from attaining the relatively high absorbance levels observed in the autoxidation process. 

**Figure 6:**
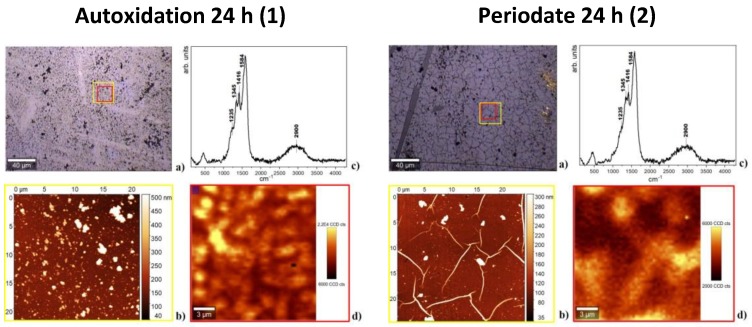
Atomic force microscopy (AFM) and micro-Raman analysis of polydopamine (PDA) films deposited at 24 h following autoxidation (1) and periodate oxidation (2). (**a**) Bright-field image of the investigated sample region collected by 20× microscope objective. (**b**) AFM image of the area indicated by the yellow square in the optical image. Average grain size: 200 nm (1), 150 nm (2). Film thickness: 100 ± 30 nm (1), 55 ± 15 nm (2). (**c**) Raman spectrum. (**d**) Micro-Raman image relative to the red sample region in the optical image.

Figure 6 shows the morphology and properties of PDA films obtained in the presence and in absence of periodate at 24 h oxidation time using AFM and micro-Raman analysis (for data at 1 h, see Supplementary Figure S2). 

Raman spectra did not reveal significant structural differences among the various samples. All spectra showed main bands at 1584, 1416, 1345, and 1235 cm^−1^ compatible with the presence of aromatic rings, while a broad band, attributable to strongly hydrogen-bonded –OH and –NH stretching vibrations, as descripted in the previous work [11], was apparent around 2900 cm^−1^. 

Atomic force microscopy analysis showed smoother and more homogeneous films by the periodate reaction. The mean estimated thickness was 55 nm, and the surface was characterized by grains of dispersed sizes and thread-like structures whose origin could not be elucidated in detail, but this was not due to adventitious or spurious objects. Micro-Raman analysis (see Figure 6(2d)) supported however the view that such protrusions were made of the same material as that of the flat sample area.

Autoxidation of dopamine led to smoother films as apparent from optical, AFM and micro-Raman images at 24 h. Dispersed grains size from 70 to 350 nm were observed for this film by AFM. This observation is consistent with previous work showing that increasing the oxygen concentration of the solution [14], or use of oxidants [15], leads to higher film uniformity and lesser roughness compared to autoxidation conditions with ambient oxygenation. This difference may be due to more efficient stacking interactions, resulting in a better packing, due to a more homogeneous composition of the reaction mixture.

Insight into the structure of the PDA samples produced by periodate oxidation versus autoxidation was then obtained by solid state ^13^C and ^15^N NMR analysis. 

**Figure 7:**
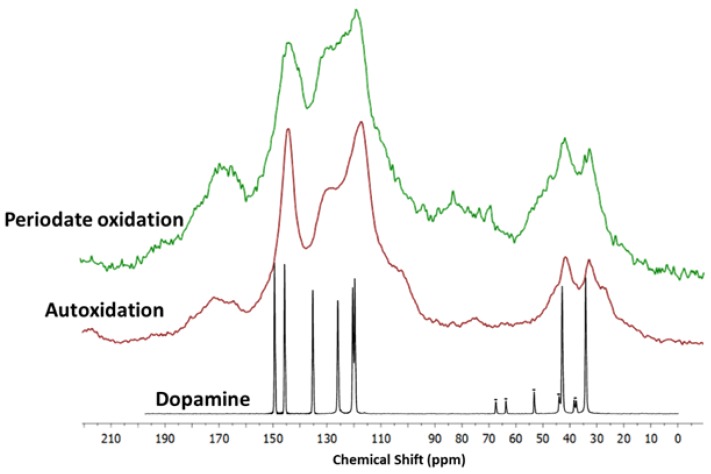
^13^C solid-state nuclear magnetic resonance (NMR) spectra of the polydopamine (PDA) samples produced by periodate oxidation (green trace) and autoxidation (red trace). The spectrum of dopamine (black trace) is reported as a reference (marked signals are due to spinning side bands).

^13^C spectra of the sample obtained by auto-oxidation and by periodate-induced oxidation showed the same main resonances. Nevertheless, some differences in the shape of aromatic, aliphatic, and carbonyl/carboxyl peaks can be evidenced (Figure 7). In particular, in the spectrum of periodate-oxidation sample a decrease of the intensity of the band at 145 ppm (OH-bearing carbon of catechol) with respect to the signal at 130 ppm (quaternary carbons) can be related to a lower content of reduced catechol moieties. This finding is further supported by the increased intensity of the carbonyl/carboxyl peak at ≈170 ppm, indicating a higher conversion of catechol to oxidized quinone groups and to some extent their oxidative fission leading to carboxyl groups in the presence of periodate [6]. Finally, comparing the signals of the aliphatic and aromatic regions, a higher relative intensity of the aliphatic bands in the spectrum of periodate oxidation sample is apparent as expected for a lower content of cyclized, indole-type units.

**^1^**^5^N NMR spectra of autoxidation and periodate oxidation samples are reported in Supplementary Figure S3. In both spectra two main resonance groups, at 30 ppm (sp^3^ hybridization) and 120–170 (sp^2^ hybridization) ppm, are observed. The complex sp^2^ band, centered at 130 ppm, is compatible with indole/pyrrole-like structures, however the poor resolution prevents a detailed assignment. The sp^3^/sp^2^ intensity ratio is comparable in both samples. 

### 3.3. Film Growth Mechanisms 

**Figure 8:**
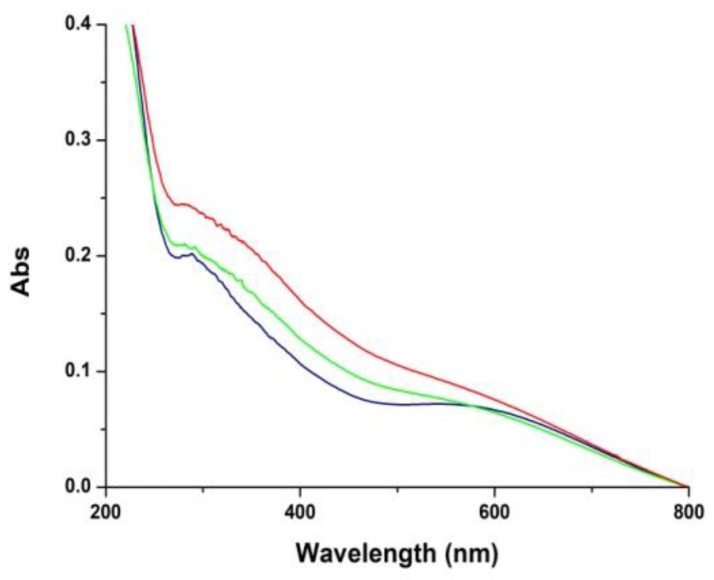
Ultraviolet–visible (UV–visible) spectra of polydopamine (PDA) coating (blue trace), and PDA coating immersed in a 0.1 mM dopamine solution at pH 9.0 for 3 (green trace) and 6 h (red trace).

In a final series of experiments the mechanisms of film growth following deposition of the initial PDA coating was investigated based on changes in UV–vis absorbance. Polydopamine films obtained by periodate oxidation of 10 mM dopamine for 1 h were extensively washed, dried and immersed into solutions of 0.1 or 1 mM dopamine at pH 9.0 (under these conditions no coating was deposited in control experiments using uncoated quartz substrates). The results showed a detectable increase in the film absorbance over 6 h (Figure 8). This observation suggests that as soon as film deposition begins, dopamine and/or other residual components, including cyclized species that are present in solution may slowly adhere to the primer layers, thus contributing to film growth by adsorption and further oxidation on the surface.

## 4. Discussion

Intense research activity into PDA film deposition over the past few years has contributed to delineate an improved picture of the underlying structural factors and features of the process, including kinetic profiles, concentration dependence, role of oxidants and amine-based additives. Very recently, a thermodynamic top-down investigation of the effects of oxidants and pH on dopamine oxidation showed that the crucial parameters in autoxidation are the pKa values of the semiquinone and the amino group in the oxidized quinone [16]. However, in most cases such advancements have not been integrated into a clear-cut mechanistic scenario, whereby several gaps have remained unsettled. The results reported in this study may contribute to provide an improved description of the basic chemical mechanisms underlying PDA film deposition and growth. Overall, previous and current data concur to indicate that film formation depends on two distinct and interrelated processes, a deposition phase controlled by adhesion mechanisms, and a growth phase involving progressive thickening of the deposited layer with alteration of its morphology. In the present study it was possible to confirm that film deposition is critically dependent on dopamine concentration, becoming almost negligible below the threshold of 1 mM dopamine (UV–vis evidence), is accelerated by quinone-forming oxidants like periodate (supporting earlier [17] and more recent work [18]) and is reinforced by the addition of long chain primary amines or diamines. 

**Figure 9:**
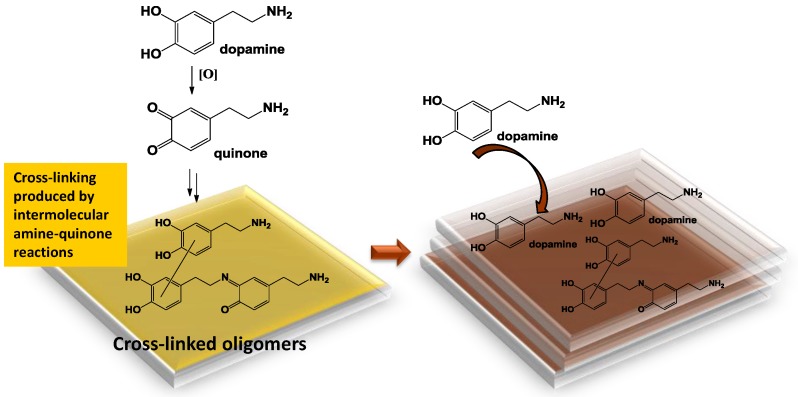
Schematic illustration of a possible interplay of polydopamine (PDA) film deposition (yellow) and growth pathways (red).

In addition, it was shown that: (a) The rapid oxidation of dopamine with periodate results in the rapid deposition of film which however do not attain the levels observed by autoxidation (UV–vis evidence); and (b) soluble monomers/oligomers can be physically adsorbed on the primer coating leading to a UV–vis detectable growth of pre-existing films. This process can be observed at a dopamine concentration of 1 mM, which does not lead to detectable coatings in the absence of the primer film. To put these observations into a rational framework of competing mechanisms it is necessary to look at amine-quinone reactions, mainly Michael-type and Schiff-base chemistry [19], as the key events triggering film deposition. In this framework, the concentration dependence of film formation can be explained by considering the evolution of dopamine quinone as a key branching point in the PDA pathway [6]. At high dopamine concentration, the quinone may be engaged with sequential bimolecular coupling processes of the dopamine-quinone or quinone-quinone type, which would direct the oxidative process toward oligomers featuring uncyclized amine groups and giving rise to adhesive cross-linked structures. Failure to observe film deposition at relatively low dopamine concentrations would then be a consequence of the prevalence of the unimolecular cyclization pathway of the quinone to give eventually DHI, which polymerizes to give insoluble eumelanin-type oligomers with a strong tendency to aggregate and very poor adhesion properties [11]. Figure 9 reports a schematic illustration of a possible interplay of film deposition and growth pathways highlighting the role of intermolecular amine quinone conjugation and cross-linking processes involved in film formation.

Although partly speculative, this scheme would well explain some observations such as the effect of periodate on thickness and roughness, the effect of long chain amines and the observed increase in film absorbance following immersion in dopamine solutions. Use of periodate ensures that all dopamine is rapidly converted to the quinone, favoring cross-linking reactions. However, while a minor amount of the material adheres to the substrate to form the primer layers, the remainder of the dopamine and other monomers would be rapidly converted to large insoluble aggregates, which would no longer contribute to the subsequent growth of the film, as observed in the slow autoxidation process. The lower roughness of the films produced by periodate oxidation compared to autoxidation is in accordance with previous findings using either higher oxygen concentration [14] or CuSO_4_/H_2_O_2_ [15], and can be attributed to the rapid conversion to quinone and generation of homogenous and more regular structures followed by deposition of PDA nanoparticles. 

The effect of long-chain diamines in promoting film formation at low dopamine concentrations can thus be attributed to coupling with the quinone, via e.g., addition or Schiff-base formation. Inclusion of long and flexible aliphatic chains into the main structural components of PDA via amine groups would account for: (a) The provision of a hydrophobic component, which is critical for underwater adhesion; (b) the inhibition of intramolecular cyclization by occupying critical positions or via Schiff-base formation; and (c) the inhibition of aggregate formation which is important for adhesion.

Work in progress is directed to validate this scheme by isolating and characterizing the primary intermediates in dopamine oxidation at high concentration to assess their adhesion properties. 

## Supplementary Materials

The following are available online at http://www.mdpi.com/2313-7673/3/3/26/s1, Figure S1: AFM analysis of the PDA film obtained in the presence of amines, Figure S2: AFM and micro-Raman analysis of PDA films obtained in the presence and in absence of periodate at 1 h, Figure S3: ^15^N spectra of samples produced by periodate oxidation and autoxidation.
