# Supplementary material for: The Chemistry of Polydopamine Film Formation: The Amine-Quinone Interplay
Source: Biomimetics (Basel). 2018 Sep 13;3(3):26. doi: 10.3390/biomimetics3030026 (PMC6352855; doi:10.3390/biomimetics3030026)
Supplement: Supplementary file 1 [file biomimetics-03-00026-s001.pdf]

Biomimetics **2018**, *3*, 26

Supplementary Materials: The Chemistry of Polydopamine Film Formation: The Amine-Quinone Interplay

Maria Laura Alfieri [1], Lucia Panzella [1], Stefano Luigi Oscurato [2], Marcella Salvatore [2], Roberto Avolio [3], Maria Emanuela Errico [3], Pasqualino Maddalena [2], Alessandra Napolitano [1] and Marco d'Ischia [1,*]

[1] Department of Chemical Sciences, University of Naples Federico II, Via Cinthia 4, I-80126 Naples, Italy; marialaura.alfieri@unina.it (M.L.A.); panzella@unina.it (L.P.); alesnapo@unina.it (A.N.)

[2] Department of Physics "Ettore Pancini", University of Naples Federico II, Via Cinthia 4, I-80126 Naples, Italy; oscurato@fisica.unina.it (S.L.O.); marcella.salvatore@fisica.unina.it (M.S.); pasmad@fisica.unina.it (P.M.)

[3] Institute for Polymers, Composites and Biomaterials, National Council of Research of Italy (IPCB-CNR), via Campi Flegrei 34, I-80078 Pozzuoli, Italy; roberto.avolio@ipcb.cnr.it (R.A.); mariaemanuela.errico@ipcb.cnr.it (M.E.E.)

* Correspondence: dischia@unina.it; Tel.: +39-081-674132

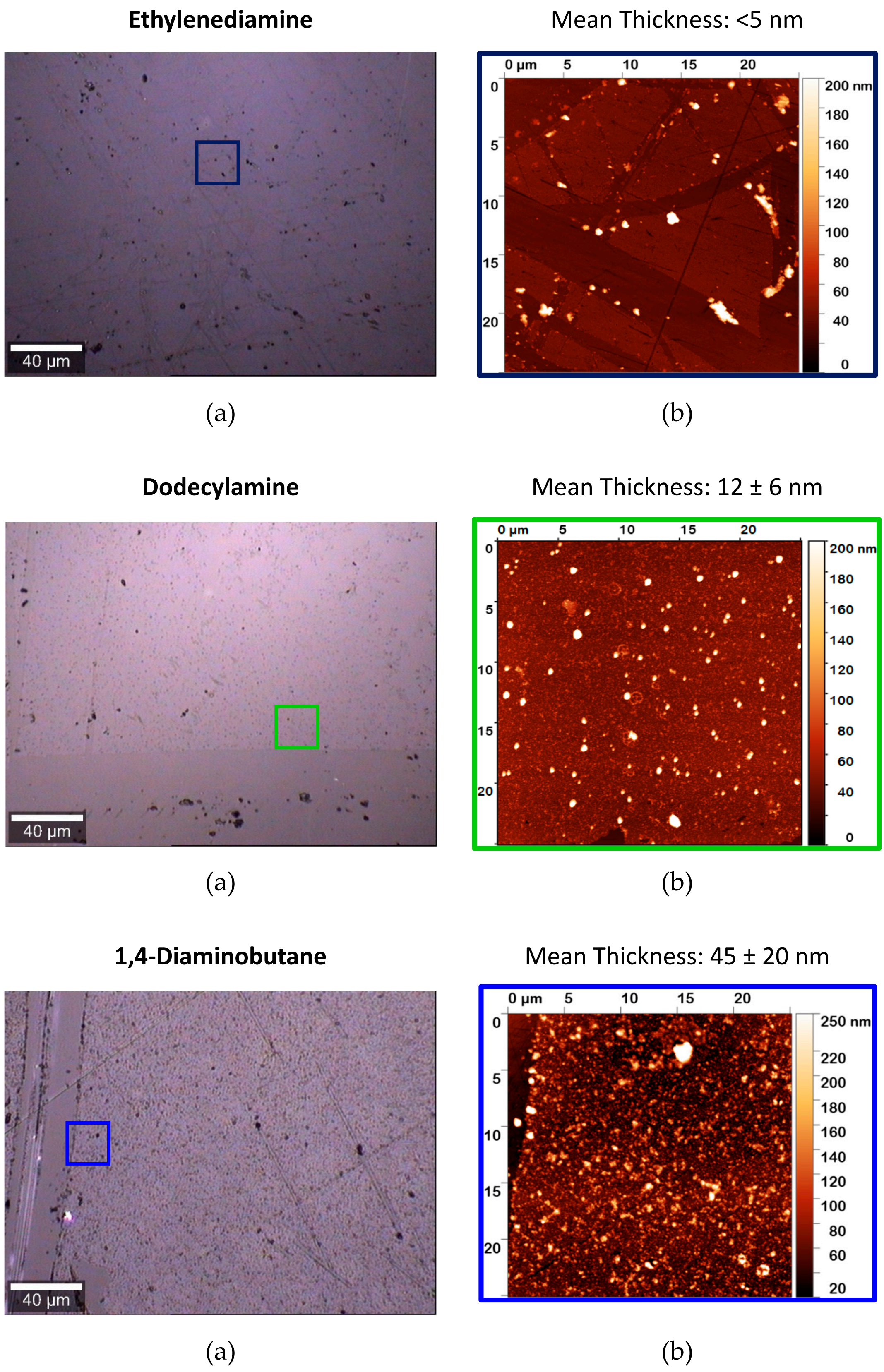

Figure S1. *Cont.*

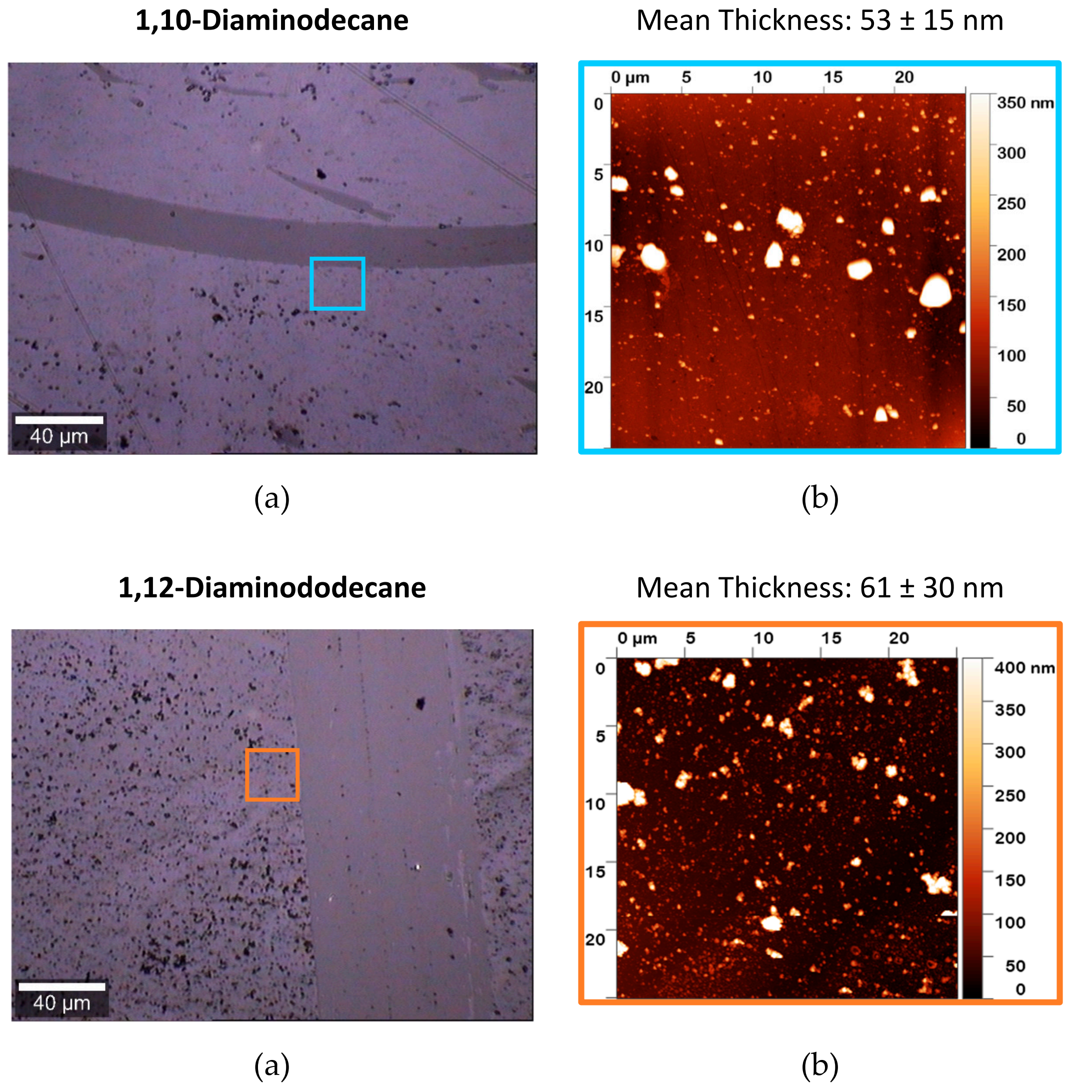

Figure S1. AFM analysis of the PDA film obtained in the presence of amines. (**a**) Bright-field image of the investigated sample region collected by 20× microscope objective. (**b**) AFM image of the area indicated by the square in the optical image.

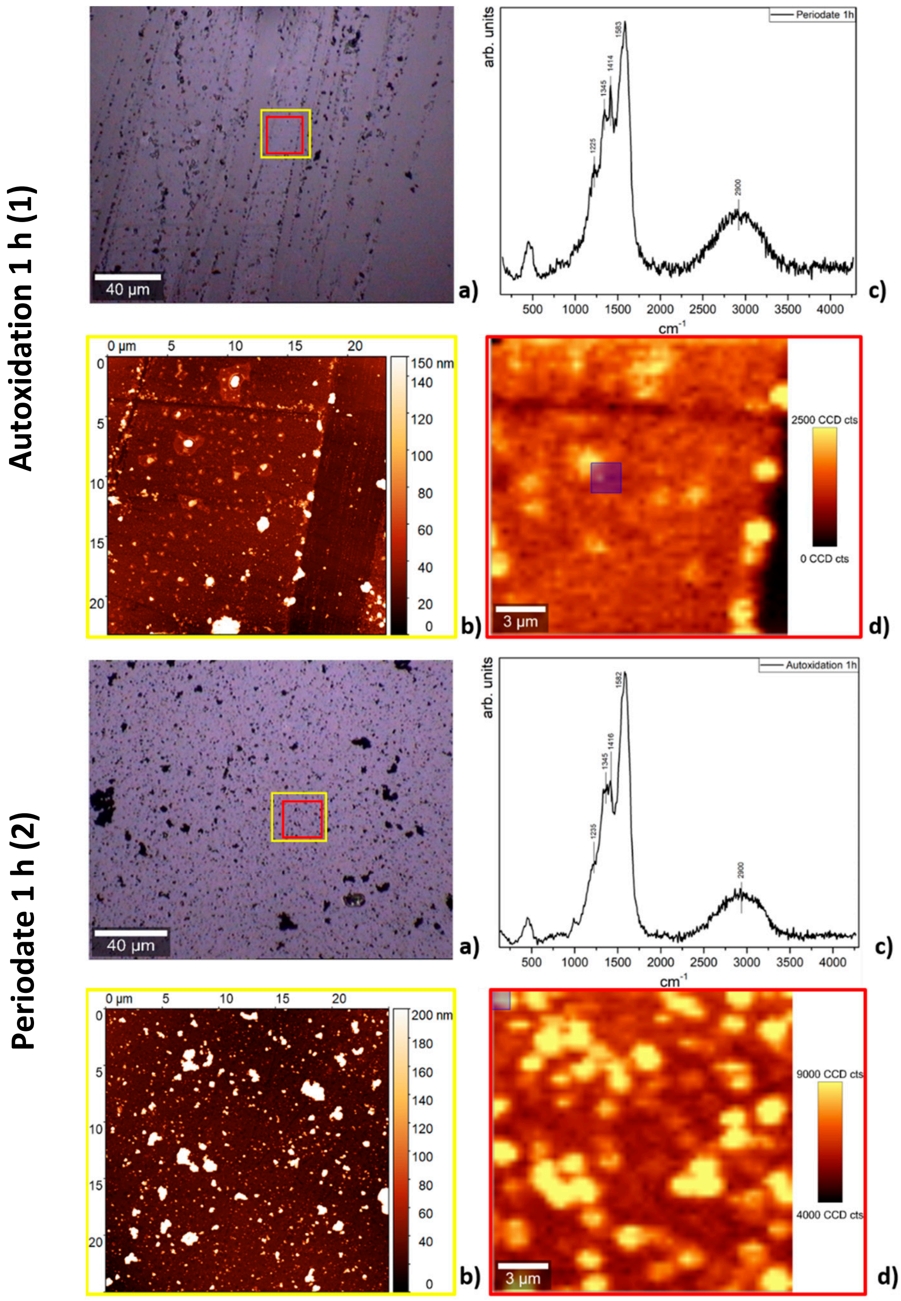

Figure S2. AFM and micro-Raman analysis of PDA films obtained in the presence and in absence of periodate at 1 h oxidation time. (**a**) Bright-field image of the investigated sample region collected by 20× microscope objective. (**b**) AFM image of the area indicated by the yellow square in the optical image. Average grain size: 60 nm (1), 100 nm (2). Film thickness: 17 ± 7 nm (1), 70 ± 15 nm (2). (**c**) Raman spectrum. (**d**) Micro-Raman image relative to the red sample region in the optical image.

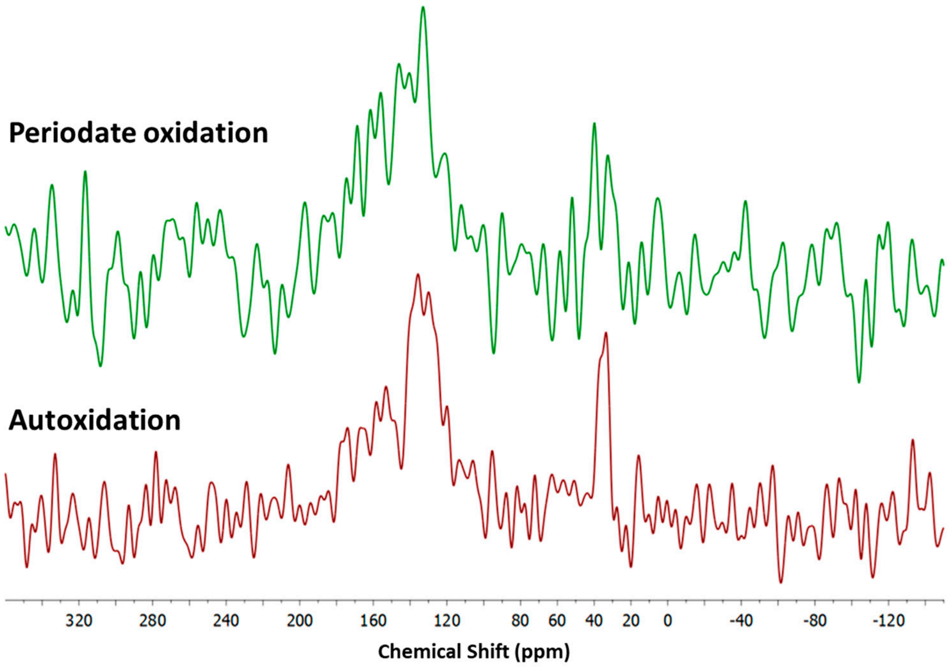

Figure S3. ^{15}N spectra of samples produced by periodate oxidation (green spectrum) and autoxidation (red spectrum).
